# Effect of Bi-Level Positive Airway Pressure (BIPAP) Ventilation on Gas Exchange, Body Mass Index, and Body Composition in Patients with Obesity Hypoventilation Syndrome

**Published:** 2019-04

**Authors:** Babak Amra, Behrooz Samadi, Forogh Soltaninejad

**Affiliations:** 1 Pulmonary Ward, Bamdad Respiratory and Sleep Research Center, Isfahan University of Medical Sciences, Isfahan, Iran; 2 Department of Internal Medicine, Medical School, Isfahan University of Medical Sciences, Isfahan, Iran.

**Keywords:** Obesity hypoventilation syndrome, Bi-level positive airway pressure ventilation, Gas exchange, Body mass index, Body composition

## Abstract

**Background:**

This study aimed to assess the outcomes of bi-level positive airway pressure (BIPAP) therapy among patients with obesity hypoventilation syndrome (OHS).

**Materials and Methods:**

In this prospective observational study, a total of 30 patients with OHS, who were subjected to BIPAP therapy, were included. The peripheral capillary oxygen saturation (SPO_2_), partial pressure of carbon dioxide (PCO_2_), body mass index (BMI), and body composition indices, including total body fat (TBF), total body protein (TBP), total body mineral (TBM), and total body water (TBW), were measured using standard procedures at baseline and one week, one month, and six months after the onset of treatment. Changes in the variables over time were evaluated using repeated measures analysis of variance (ANOVA). The correlation between changes in the body composition indices and changes in gas exchange was also assessed by Pearson’s correlation coefficient test at three time points from the baseline.

**Results:**

The results revealed that all study variables, except for TBF, changed significantly during the study in both males and females (P<0.001). There was a significant positive association between changes in PCO_2_ and changes in TBM after six months (r=0.4, P<0.05), whereas a significant inverse correlation was found between changes in PCO_2_ and changes in TBW after six months (r=−0.39, P<0.05). However, no significant correlation was found between changes in gas exchange and changes in BMI or other body composition indices after six months.

**Conclusion:**

The present results indicated no significant association between the improvement of gas exchange and changes in BMI, TBP, and TBF during the study. However, further large-scale studies are required to examine the effects of BIPAP therapy on body composition in patients with OHS.

## INTRODUCTION

One of the most important obesity-related diseases is obesity hypoventilation syndrome (OHS), which is associated with high morbidity and mortality. OHS is defined as daytime hypercapnia (PCO_2_) in a patient with a body mass index (BMI) ≥30 kg/m^2^ and sleep-disordered breathing in the absence of other causes of hypoventilation (1–3). Although the exact prevalence of OHS in the general population is not well established, the increased prevalence of obesity may lead to the increased prevalence of this syndrome. Also, the pathophysiology of this disease is not fully understood. However, evidence suggests that it may result from sleep-disordered breathing, as well as impairment of respiratory mechanics, central respiratory control, neurohormonal responses and neurohormonal interactions (2, 4).

Early diagnosis and treatment of OHS are essential, considering the low quality of life of patients, high healthcare expenses, and comorbidities (5–7). There are several therapeutic procedures available for OHS, including weight loss surgery, tracheostomy, pharmacotherapy, and positive airway pressure (PAP) therapy (5). The treatment of choice for OHS is PAP, which usually involves continuous positive airway pressure (CPAP) and bi-level positive airway pressure (BIPAP) (8). Nevertheless, there is no large-scale randomized controlled clinical trial, showing which type of PAP is optimal for the treatment of patients with OHS.

It has been suggested that BIPAP therapy can be used in patients with CPAP failure and OHS, associated with acute-on-chronic respiratory failure (ACRF), without obstructive sleep apnea (9). Several studies have indicated that BIPAP therapy improves both short- and long-term clinical outcomes in patients with OHS. The results of these studies have revealed that BIPAP therapy leads to significant improvements in gas exchange, sleep quality, and survival of OHS patients (10, 11). However, none of these studies have investigated body composition indices as the outcomes of BIPAP therapy.

In this prospective observational study, we aimed to determine the effect of BIPAP therapy on gas exchange, BMI, and body composition indices and to explore the association of gas exchange variables with BMI and body composition indices in patients with OHS.

## MATERIALS AND METHODS

### Study design and subjects

In this prospective observational study, patients with a confirmed diagnosis of OHS (i.e., BMI >30 kg/m^2^, diurnal PCO_2_>45 mmHg, and lack of hypoventilation secondary to other etiologies, such as neurologic, neuromuscular, and metabolic disorders) and favorable compliance with BIPAP therapy were recruited from Bamdad Respiratory and Sleep Research Center, affiliated to Isfahan University of Medical Sciences (Isfahan, Iran) during 2016–2018. The Research Ethics Committee of Isfahan University of Medical Sciences approved the study protocol (IR.MUI.REC.1396.3.186). On the other hand, patients with opium addiction, other conditions associated with hypoventilation (e.g., obstructive pulmonary disease, heart failure, and pregnancy), and use of weight-loss medications or corticosteroids were excluded from the study.

### Body composition analysis

Body composition indices, including total body protein (TBP), total body fat (TBF), total body mineral (TBM), and total body water (TBW), as well as BMI, were analyzed using a bioimpedance analyzer (Maltron International Ltd., Rayleigh, Essex, UK) at baseline and one week, one month, and six months after the onset of treatment. Also, peripheral capillary oxygen saturation (SPO_2_) and partial pressure of carbon dioxide (PCO_2_) were measured, based on standard protocols, using pulse oximetry and capnography, respectively.

### Statistical analysis

Continuous and categorical data are presented as mean±SD and frequency (percentage), respectively. The normality of continuous data was evaluated using Kolmogorov-Smirnov test and Q-Q plot. Repeated measures analysis of variance (ANOVA) was also used for assessing the mean changes of study variables over time. The assumption of sphericity for repeated measures ANOVA was examined using Mauchly’s test, and if violated, the multivariate approach was adopted. Also, independent samples t-test was used for comparing the mean values of study variables at each time point between male and female participants. The correlation between changes in BMI and body composition indices and changes in PCO_2_ and SPO_2_ was assessed at three time points from the baseline, using Pearson’s correlation test. All statistical tests were conducted using SPSS.

## RESULTS

**Figure 1.**
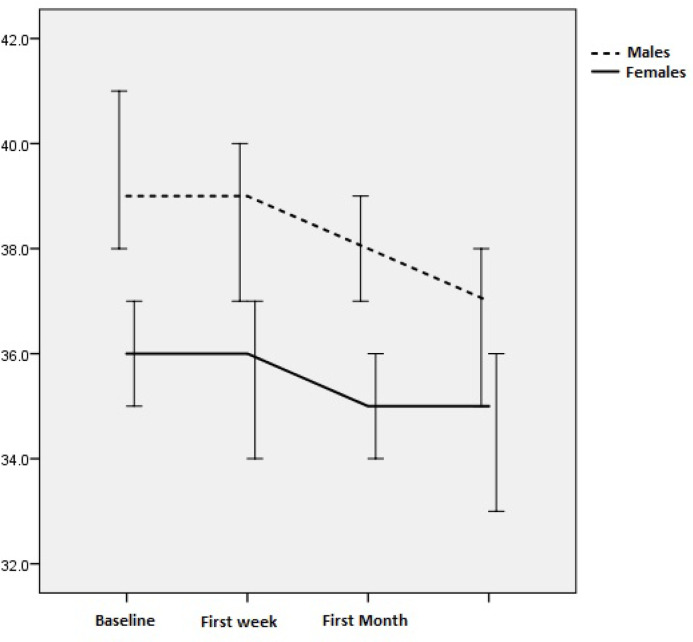
BMI changes in patients with obesity hypoventilation syndrome during study period

A total of 30 patients with OHS (20 males and 10 females), with the mean age of 58.03±10.37 years, were included in this prospective observational study. Data regarding SPO_2_, PCO_2_, BMI, and body composition indices at baseline and one week, one month, and six months after the onset of treatment are summarized in Table 1. All variables, except for TBF, changed significantly over time in the total sample and both males and females (P<0.001). The mean values of TBP, TBM, TBW, and SPO_2_ increased, while BMI (Figure 1) and PCO_2_ decreased during the study. The total sample, as well as male and female subjects, showed the same pattern in terms of changes in the study variables (Table 1).

**Table 1.** Mean values of study variables during the study period^*^

**BMI (kg/m^2^)**	**Baseline**	**1^st^ week**	**1^st^ month**	**6^th^ month**	**P value**
Total	38.86 ±6.14	38.13 ±5.78	37.49 ±5.74	36.57 ±5.42	<0.001
Males	39.99 ±6.76	39.15 ±6.22	38.49 ±6.14	37.25 ±5.92	<0.001
Females	36.59 ±4.07	36.09 ±4.36	35.48 ±4.45	35.22 ±4.19	0.01
P value	0.16	0.18	0.18	0.34	
**Total body fat (kg)**					
Total	46.94 ±11.65	45.64 ±10.71	46.10 ±10.61	45.57 ±10.76	0.1
Males	48.74 ±12.38	46.41 ±11.63	47.25 ±11.35	47.00 ±11.51	0.2
Females	43.34 ±8.25	44.10 ±8.96	43.80 ±9.05	42.70 ±8.95	0.61
P value	0.24	0.59	0.41	0.31	
**Total body protein (kg)**					
Total	4.79 ±1.91	6.63 ±1.96	8.15 ±2.03	9.87 ±2.34	<0.001
Males	4.73 ±1.76	6.65 ±1.71	8.05 ±1.90	9.63 ±1.95	<0.001
Females	4.89 ±2.30	6.57 ±2.50	8.35 ±2.37	10.34 ±3.05	<0.001
P value	0.83	0.91	0.71	0.44	
**Total body mineral (kg)**					
Total	2.10 ±0.90	3.09 ±0.97	4.09 ±0.98	5.08 ±0.97	<0.001
Males	2.26 ±0.77	3.24 ±0.93	4.24 ±0.93	5.22 ±0.93	<0.001
Females	1.79 ±0.98	2.80 ±1.03	3.785 ±1.05	4.80 ±1.03	<0.001
P value	0.17	0.25	0.23	0.27	
**Total body water (liter)**					
Total	45.10 ±9.59	60.72 ±11.13	71.17 ±11.33	83.50 ±9.89	<0.001
Males	47.04 ±9.91	61.88 ±12.87	71.61 ±12.72	83.11 ±11.21	<0.001
Females	41.20 ±7.96	58.40 ±6.75	70.30 ±8.43	84.30 ±6.98	<0.001
P value	0.12	0.43	0.77	0.76	
**O_2_ saturation (%)**					
Total	83.87 ±2.40	86.97 ±2.47	90.57 ±2.57	93.43 ±2.18	<0.001
Males	83.50 ±2.52	86.80 ±2.59	90.75 ±2.40	93.60 ±2.50	<0.001
Females	84.60 ±2.07	87.30 ±2.31	90.20 ±2.97	93.10 ±1.37	<0.001
P value	0.24	0.61	0.59	0.56	
**PCO_2_(mmHg)**					
Total	68.60 ±6.11	60.07 ±7.03	53.20 ±7.09	45.17 ±5.57	<0.001
Males	68.30 ±6.35	60.20 ±7.35	54.00 ±7.02	45.50 ±5.65	<0.001
Females	69.20 ±5.86	59.80 ±6.70	51.60 ±7.34	44.50 ±5.62	<0.001
P value	0.71	0.89	0.39	0.65	

* Data are presented as mean±standard deviation, p-values resulted from repeated measures ANOVA

Pearson’s correlation test demonstrated an inverse significant correlation between changes in SPO_2_ after six months and changes in TBP after the first week (r=−0.4, P<0.05) and the first month (r=−0.37, P<0.05) (Table 2). On the other hand, a marginally significant positive correlation was found between changes in PCO_2_ after six months, and changes in TBP after one month (r=0.36, P<0.1). There was also a significant positive correlation between changes in PCO_2_ after six months and changes in TBM after one week (r=0.45, P<0.001), one month (r=0.43, P<0.05), and six months (r=0.40, P<0.05).

**Table 2.** Pearson’s correlation coefficients between the changes from baseline (Δ) for BMI and body composition measurements with changes of arterial blood gas measurements from baseline

			**BMI**			**Fat**			**Protein**			**Total body water**			**Mineral**		

			1^st^ week	1^st^ month	6^th^ month	1^st^ week	1^st^ month	6^th^ month	1^st^ week	1^st^ month	6^th^ month	1^st^ week	1^st^ month	6^th^ month	1^st^ week	1^st^ month	6^th^ month
**PCO_2_**	1^st^week	r	− 0.33^*^	− 0.29	− 0.25	− 0.08	− 0.07	− 0.03	0.04	0.02	− 0.14	0.14	0.01	0.02	0.14	0.14	0.11
	1^st^ month	r	− 0.35^*^	− 0.36^*^	− 0.34^*^	− 0.13	− 0.10	− 0.10	0.14	0.08	− 0.07	− 0.03	− 0.11	− 0.26	0.31	0.31^*^	0.28
	6^th^ month	r	− 0.13	− 0.20	− 0.09	− 0.25	− 0.06	− 0.02	0.22	0.36^*^	0.12	− 0.01	− 0.20	− 0.39^**^	0.45^**^	0.43^**^	0.40^**^
**O_2_ saturation**	1^st^week	r	0.39^**^	0.45^**^	0.26	− 0.15	− 0.08	0.07	− 0.21	− 0.29	−0.28	− 0.16	−0.28	− 0.11	0.08	0.10	0.09
	1^st^ month	r	0.43^**^	0.43^**^	0.25	− 0.17	− 0.27	− 0.15	− 0.36^**^	− 0.33^*^	− 0.25	− 0.06	− 0.10	0.10	− 0.08	− 0.03	− 0.05
	6^th^ month	r	0.28	0.43^**^	0.25	− 0.11	− 0.24	− 0.18	− 0.40^**^	− 0.37^**^	− 0.28	− 0.07	0.06	0.22	− 0.27	− 0.22	− 0.23

* indicates p <0.1

** indicates p<0.05

*** indicates p<0.01

The present results revealed an inverse correlation between changes in PCO_2_ after six months and changes in TBW after six months (r=−0.39, P<0.05). A significant positive correlation was found between changes in BMI and changes in SPO_2_, whereas a significant negative correlation was observed between changes in BMI and changes in PCO_2_. However, the correlation between these variables was not significant after six months.

## DISCUSSION

OHS is a major respiratory disease associated with obesity. PAP therapy, characterized by CPAP and BIPAP ventilation modes, is considered the first-line treatment for OHS. However, there is no robust evidence regarding the best ventilation mode for these patients (12). The results of this prospective study showed that BIPAP therapy improved gas exchange, BMI, and some body composition indices in patients with OHS. However, no significant correlation was found between changes in PCO_2_ and SPO_2_ and changes in BMI, TBF, and TBP six months after treatment, suggesting that BIAP improves gas exchange in patients with OHS, regardless of changes in the body composition. To the best of our knowledge, this is the first study assessing body composition as a treatment outcome in OHS patients.

So far, only a few studies have investigated the association between changes in BMI and gas exchange in OHS patients, receiving positive pressure ventilation. In this regard, Llano et al. showed that hypercapnia and hypoxemia significantly improved among patients with OHS following nasal intermittent positive pressure ventilation (NPPV) in the 50-month follow-up; however, these patients exhibited a non-significant change in BMI (11). This result is consistent with another study regarding the effects of NPPV on pulmonary function and gas exchange, which showed a significant improvement in gas exchange, regardless of weight change in patients with OHS (13).

The present results also showed that changes in PCO_2_ and SPO_2_ had no significant correlations with changes in BMI after six months. However, our results indicated a significant positive association between changes in BMI and changes in O_2_ saturation during the first month of treatment; nonetheless, the association became non-significant after six months. On the contrary, Priou et al. observed that baseline BMI was significantly correlated with PCO_2_ in patients with OHS after six months of NPPV therapy; however, they did not assess the association between changes in BMI during the study and changes in gas exchange. They concluded that NPPV treatment might be less effective for patients with severe obesity (10). Differences in the baseline characteristics of the study population and design may explain the differences in the results of these studies. Therefore, further large-scale clinical trials are warranted to assess the effect of BIPAP therapy on changes in BMI and to determine its correlation with changes in gas exchange.

The current literature suggests the favorable impact of weight loss on improving the symptoms of OSH. In this regard, the results of a study by Murphy et al. showed that nocturnal ventilatory support for OHS patients increases their physical activity and weight loss, which are associated with the improvement of sleep-disordered breathing (14). Our results also indicated that BMI significantly decreased after six months in OHS patients following BIPAP treatment. It seems that BMI improvement in OHS patients in the current study was related to lifestyle modifications, such as increased physical activity and adherence to a healthy diet, which could be directly associated with BIPAP therapy or result from the educational programs.

The current study had some limitations. The lack of a control group and the limited number of recruited patients are the major limitations of this study. Also, the short follow-up period is another limitation of this study, which precludes long-term evaluation of treatment outcomes in OHS patients, who received BIPAP ventilation.

In conclusion, the present study suggested that BIPAP ventilation is an effective treatment option for patients with OHS, as it can improve gas exchange and body composition indices. However, further large-scale, long-term randomized controlled clinical trials are required to determine whether body composition is a clinical outcome of BIPAP therapy.
